# Human Mesenchymal Stem Cells Prolong Survival and Ameliorate Motor Deficit through Trophic Support in Huntington's Disease Mouse Models

**DOI:** 10.1371/journal.pone.0022924

**Published:** 2011-08-05

**Authors:** Yuan-Ta Lin, Yijuang Chern, Che-Kun James Shen, Hsin-Lan Wen, Ya-Chin Chang, Hung Li, Tzu-Hao Cheng, Hsiu Mei Hsieh-Li

**Affiliations:** 1 Institute of Biochemistry and Molecular Biology, National Yang-Ming University, Taipei, Taiwan; 2 Institute of Molecular Biology, Academia Sinica, Taipei, Taiwan; 3 Institute of Biomedical Sciences, Academia Sinica, Taipei, Taiwan; 4 Department of Life Science, National Taiwan Normal University, Taipei, Taiwan; University of Sao Paulo – USP, Brazil

## Abstract

We investigated the therapeutic potential of human bone marrow-derived mesenchymal stem cells (hBM-MSCs) in Huntington's disease (HD) mouse models. Ten weeks after intrastriatal injection of quinolinic acid (QA), mice that received hBM-MSC transplantation showed a significant reduction in motor function impairment and increased survival rate. Transplanted hBM-MSCs were capable of survival, and inducing neural proliferation and differentiation in the QA-lesioned striatum. In addition, the transplanted hBM-MSCs induced microglia, neuroblasts and bone marrow-derived cells to migrate into the QA-lesioned region. Similar results were obtained in R6/2-J2, a genetically-modified animal model of HD, except for the improvement of motor function. After hBM-MSC transplantation, the transplanted hBM-MSCs may integrate with the host cells and increase the levels of laminin, Von Willebrand Factor (VWF), stromal cell-derived factor-1 (SDF-1), and the SDF-1 receptor Cxcr4. The p-Erk1/2 expression was increased while Bax and caspase-3 levels were decreased after hBM-MSC transplantation suggesting that the reduced level of apoptosis after hBM-MSC transplantation was of benefit to the QA-lesioned mice. Our data suggest that hBM-MSCs have neural differentiation improvement potential, neurotrophic support capability and an anti-apoptotic effect, and may be a feasible candidate for HD therapy.

## Introduction

Huntington's disease (HD) is an autosomal dominant inherited neurodegenerative disorder for which there is currently no effective treatment. It is caused by an unstable expansion mutation of a naturally occurring trinucleotide (CAG) repeat in exon 1 of the *IT15* gene on chromosome 4p16.3 that encodes a ubiquitously expressed 350-kDa protein named huntingtin. The disorder is characterized by intellectual decline, movement disorders and behavioral changes [1], [2] that lead to severe debilitation and death, usually within 15–20 years. The neuropathological changes in HD are selective and progressive degeneration of striatal GABAergic medium spiny projection neurons [3] accounts for most of the clinical features. Currently, there is no proven medical therapy to alleviate the onset or progression of Huntington's disease [4].

The clinical uses of cell replacement therapy in neurodegenerative diseases have been investigated for the last 20 years. Although the procedures are theoretically feasible, some limitations of the therapy still give cause for concern. The transplantation of fetal striatal tissue to the striatum to modify HD progression in humans has been investigated, and some favorable effects have been found [5], [6]. Transplanted fetal neurons can lead to functional benefit and repair [5], and the transplanted cells remain viable in the human neostriatum for long periods of time [6]. However, there are still many unsolved difficulties associated with the transplantation of human fetal striatal tissue for therapy in HD such as ethical arguments, viability of tissue source, limitations on tissue acceptance, the high risk of rejection and concerns about contamination and heterogeneity of the tissues [7].

The use of renewable and expandable bone marrow-derived mesenchymal stem cells (BM-MSCs) circumvents many of the practical and ethical problems associated with the use of human fetal tissue. BM-MSCs are easy to acquire, have self-renewing properties, expand rapidly, and may differentiate into all of the major cell types in the central nervous system [8]. BM-MSCs can also be harvested directly from patients, with the resulting autologous transplants avoiding the risk of immune rejection [9]. Transplanted BM-MSCs have a reduced risk of tumor formation and are able to differentiate into neuronal or glial lineages and provide functional improvement in the central nervous systems (CNS) of rodents with Parkinson's disease [10] and other neurodegenerative disorders [11], [12]. We and others have demonstrated that intracerebrally transplanted bone marrow-derived stem cells can migrate to damaged brain areas and improve neuronal function and architecture in stroke animal models [8], [13]. Furthermore, the function of neurogenic effects of human multipotent stromal cells (hMSCs) in HD mouse models had been demonstrated [14]. Therefore, MSCs may provide an alternative cell source for transplantation therapy in HD; however, the possible mechanisms involving in MSCs transplantation are still unclear. In this study, we demonstrated that hBM-MSC transplantation may have beneficial effects by increasing neurogenesis, attracting neural stem-cell migration, enhancing SDF-1 expression, and decreasing apoptosis in mouse models of HD.

## Results

### hBM-MSCs May Differentiate and Survive in C57/B6 Mice

First, we investigated whether hBM-MSCs expressed neuronal markers *in vitro*. Our results showed that GFAP (a marker for astrocytes), NeuN (a marker for neurons), and DARPP-32 (a marker of striatal medium-sized spiny projection neurons) were all not detected in the hBM-MSCs before transplantation into mouse striata.

**Figure 1:**
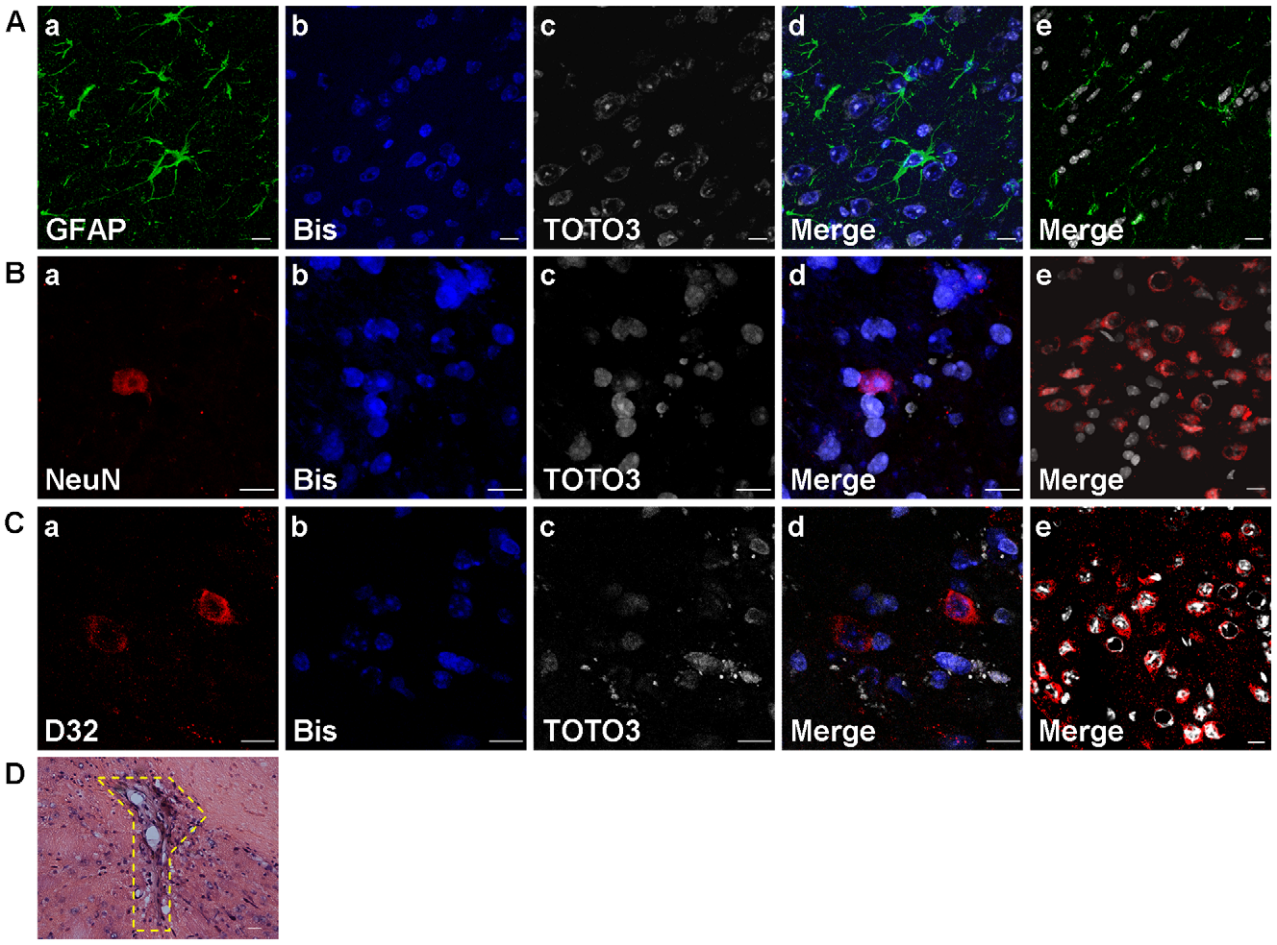
Transplanted hBM-MSCs differentiated and survived in wild-type C57/B6 mice. (A–C) Confocal laser microscopic analysis of immunofluorescence labeled striatal sections in hBM-MSCs transplanted wild-type C57/B6 mice, 8 weeks after striatal transplantation with hBM-MSCs. Transplanted hBM-MSCs pre-labeled with bisbenzimide (b; blue) or labeled with TOTO3 (c; white) were immunostained against various cell markers, including GFAP (A-a; green), NeuN (B-a; red), and DARPP-32 (C-a; red). Corresponding merged images are shown accordingly (d). Images from the sham-control group (e) are stained with the same cell markers as hBM-MSC-transplant group (d). Scale bar = 10 µm. (D) After 17 months, hematoxylin and eosin staining of brain transplanted with hBM-MSCs showed no turmorigenesis in the transplantation region (yellow dotted line demarcates the involved region). Scale bar = 20 µm.

Next, we investigated the fate of hBM-MSCs after transplantation in C57/B6 mice. We used bisbenzimide labeling [15] and immunofluorescence with cell type-specific markers to trace and determine the cell types of differentiated hBM-MSCs in the striatum of C57/B6 mice. Eight weeks after hBM-MSC transplantation, confocal microscope images showed some bisbenzimide-labeled cells co-expressing GFAP (Fig. 1A; a–d), NeuN (Fig. 1B; a–d), and DARPP-32 (Fig. 1C; a–d) throughout the transplantation region in the striatum. We also used TOTO3 dye (Fig. 1; A-c, B-c & C-c) to confirm the positions of cell nuclei, and found the TOTO3 signal in many cells was colocalized with the bisbenzimide signal, demonstrating the differentiation of hBM-MSCs after transplantation. Such cell differentiation was not obvious in the sham-control group (Fig. 1; A-e, B-e & C-e).

Tumorigenesis is an important consideration in stem cell transplantation. To investigate the tumorgenetic potential of hBM-MSCs, we transplanted hBM-MSCs into the striatum of C57/B6 mice and followed them for at least 17 months. Seventeen months after hBM-MSC transplantation, there was no turmorigenesis in the surgical region as assessed by hematoxylin and eosin staining (Fig. 1D). These results suggest that hBM-MSCs could survive and differentiate successfully within the transplanted mouse striatum.

### hBM-MSC Transplantation Improves Striatum Volume, Rotarod Performance, and Survival Rates after QA-induced Excitotoxicity

**Figure 2:**
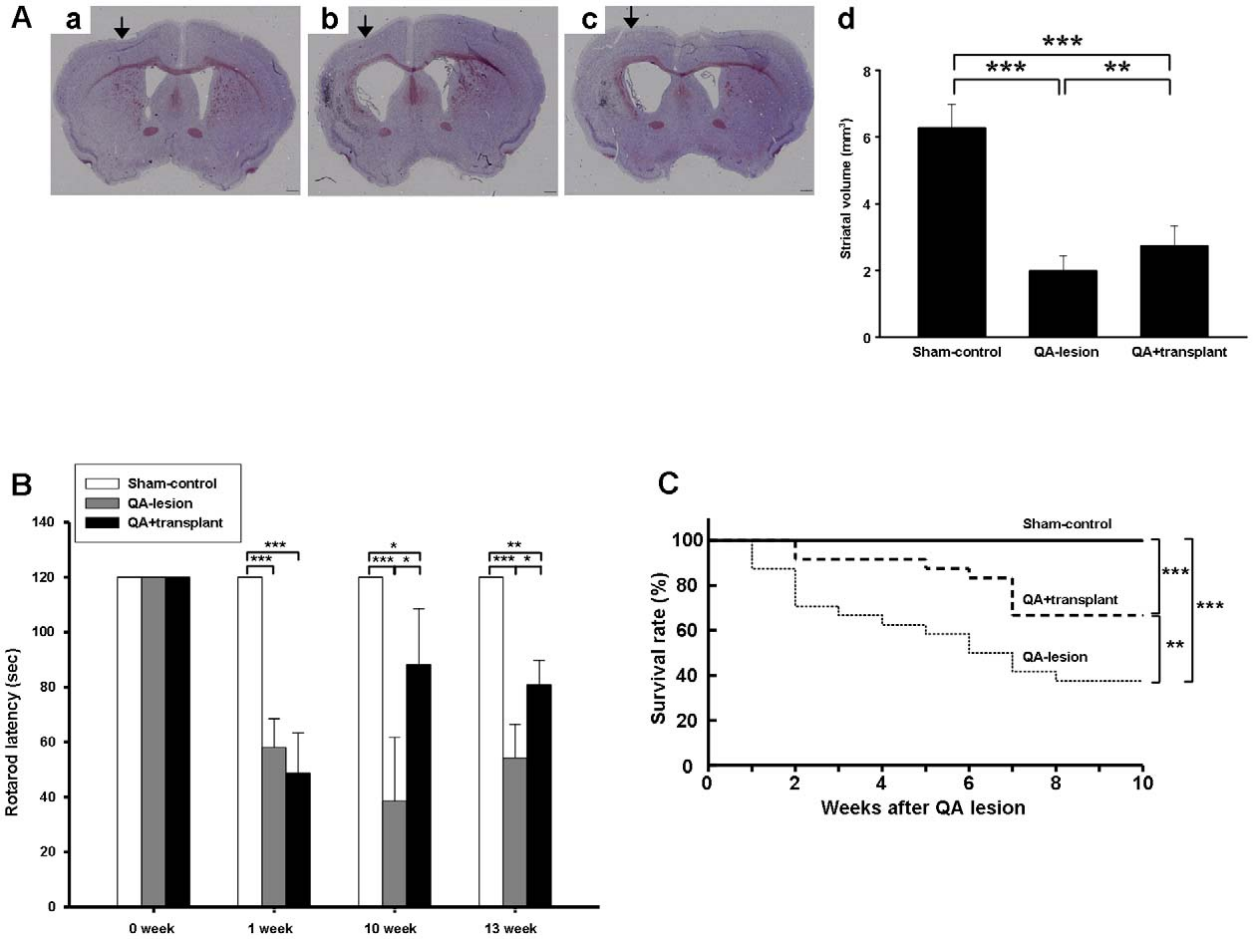
Transplanted hBM-MSCs improved striatal volumes, motor function, and survival rates in QA−lesioned mice. (A) Photomicrographs show the difference in striatal volumes in unilateral lesion mice. There was no striatal atrophy in the sham-control group (a), but the striatal volumes in the lesion side of QA−lesioned (b) and QA+hBM-MSC-transplant (c) groups were significantly decreased compared with the contralateral side. Arrows indicate surgery sites in each group. Scale bar = 400 µm. (d) Quantification of striatal volumes show increased volumes in the QA+hBM-MSC-transplant group. (B) Rotarod performance of mice was tested over 2 min duration at 30 r.p.m. Sham-control mice exhibited normal motor function, but QA−lesioned and QA+transplant groups showed decreased motor function at 1, 10, and 13 weeks after QA lesioning. Ten and 13 weeks after QA lesioning, the QA+transplant group exhibited better motor function than the QA−lesioned group. (C) Survival rates after QA lesioning. Twenty-four mice were all alive in the sham-control group, whereas 9/24 mice (37.5%) were alive in the QA−lesioned group and 16/24 (66.7%) were alive in the QA+transplant group 10 weeks after surgery. Error bars represent SD, and *P<0.05, ** *P*<0.001, *** *P*<0.0001.

We used the QA-lesioned mouse model to evaluate the effects of hBM-MSC transplantation. Ten weeks after surgery, striatum volumes were not changed in the sham-control mice (Fig. 2A; a), but were obviously decreased in the QA-lesioned (Fig. 2A; b) and QA+transplant groups (Fig. 2A; c). However, striatum volumes in QA+transplant group were partially recovered in comparison with QA-lesioned group (Fig. 2A; d). The striatum volume of the wild-type sham-control was approximately 6.3 mm^3^, while in the QA−lesioned and QA+transplant groups striatum volume was 2.0 mm^3^ and 2.7 mm^3^, respectively. We then used rotarod performance as an indicator of motor function in mice to estimate the effects of hBM-MSCs. One week after QA lesioning, both the QA−lesioned and QA+transplant groups showed an obvious reduction in rotarod performance compared to normal mice. The motor function of the QA+transplant group recovered more rapidly than the QA−lesioned group and was significantly different from 10 weeks until at least 13 weeks (Fig. 2B) after transplantation. The survival rate of the QA+transplant mice during the 10 weeks after QA lesioning was significantly higher than that of the QA−lesioned mice (Fig. 2C), suggesting that hBM-MSC transplantation could elicit behavioral and survival recovery after QA lesioning.

### hBM-MSCs Improve Cell Proliferation and Differentiation after Transplantation in QA−lesioned Mice

**Figure 3:**
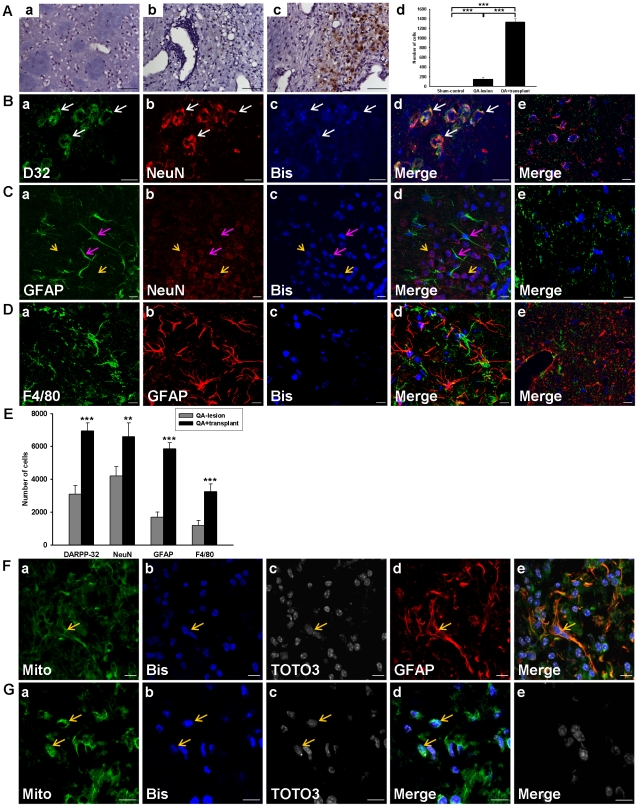
Transplanted hBM-MSCs improved cell proliferation and differentiation in QA−lesioned mice. (A) Photomicrographs showing the presence of BrdU-labeled cells throughout the transplanted hemisphere. Only rare BrdU signals were detected in the sham-control group (a) and the QA−lesioned group (b), but many BrdU-labeled cells (dark brown) were observed in the QA+hBM-MSC-transplant striatum (c) 10 weeks after transplantation. Scale bar = 50 µm. (d) Quantification of BrdU-labeled cells showed numerous BrdU-labeled cells in the QA+hBM-MSC-transplant group. (B–D) Confocal laser microscopic analysis of immunofluorescence labeled striatal sections in QA−lesioned and hBM-MSCs transplanted mice, 8 weeks after a striatal lesioning with QA. Transplanted hBM-MSCs pre-labeled with bisbenzimide (c; blue) were immunostained against cell markers DARPP-32 (B-a; green), NeuN (B-b & C-b; red), GFAP (C-a; green & D-b; red), and F4/80 (D-a; green). Corresponding merged images are shown accordingly (d). Images from the QA−lesioned group (e) are stained with the same cell markers as QA+transplant group (d) except that the bisbenzimide labeling was substituted with DAPI staining. Arrows indicate colocalization of hBM-MSCs and cell markers, white for DARPP32, pink for GFAP, and yellow for NeuN. Scale bar = 10 µm. (E) Quantitation of DARPP-32, NeuN, GFAP, and F4/80-positive cells in the hemispheres of striata from QA+transplant and QA−lesioned groups. Error bars represent SD, and ** *P*<0.001, *** *P*<0.0001. (F–G) Confocal laser microscopic analysis of immunofluorescence labeled striatal sections in QA−lesioned and hBM-MSC transplant mice, 8 weeks (F) and 16 weeks (G) after a striatal lesioning with QA. Transplanted hBM-MSCs pre-labeled with bisbenzimide (b; blue) and co-labeled with TOTO3 (c; white) were immunostained against human mitochondria marker (a; green) or GFAP (d; red). Corresponding merged images are shown accordingly (F-e & G-d). Images from the QA-lesioned group (G-e) are stained with the same cell markers as QA+transplant group (G-d). Arrows show the colocalization of hBM-MSCs and cell markers. Scale bar = 10 µm.

To monitor the effects of hBM-MSCs after transplantation, experimental mice were injected subcutaneously with BrdU (50 mg/kg) once-a-day for 7 days before being sacrificed at different time-points. Sixteen weeks after QA lesioning, BrdU-immunoreactive cells were rarely detected in the sham-control (Fig. 3A; a) and few were detected in the QA−lesioned mice (Fig. 3A; b); however, BrdU-immunoreactive cells were abundant in the QA+transplant group (Fig. 3A; c). The quantitative results clearly show a significantly higher number of BrdU-immunoreactive cells in the QA+transplant group than in sham-control and the QA−lesioned groups (Fig. 3A; d). In addition, the phenomenon of cell proliferation could last for at least 16 weeks after QA lesioning (data not shown), which revealed that hBM-MSC transplantation increased cell proliferation in the striatum region of QA+transplant mice.

Next, we determined the cell types and numbers of differentiated hBM-MSCs surviving in the QA impaired striatum using the same approach as used above with the C57/B6 mice. Eight weeks after hBM-MSC transplantation, confocal microscope images showed a small number of bisbenzimide-labeled cells in the QA−lesioned striatum co-expressing DARPP-32 (Fig. 3B; a–d). Furthermore, more GFAP- or NeuN-expressing cells (Fig. 3C; a–d) were seen throughout the QA impaired striatum, and a large number of GFAP-expressing cells which were not colocalized with the bisbenzimide signals surrounded the core of the QA lesion. In addition, many cells expressing F4/80 (a marker for microglia) were found near the GFAP-expressing cells (Fig. 3D; a–d). Such cell differentiation and proliferation was seldom detected in the QA−lesioned group (Fig. 3; B-e, C-e & D-e). The quantitative results show a significantly higher number of DARPP-32, NeuN, GFAP, and F4/80 positive cells in the QA+transplant group than in the QA−lesioned group (Fig. 3E).

To demonstrate that the transplanted hBM-MSCs were actually alive, we used an anti-human mitochondria antibody to trace the hBM-MSCs. We observed that cells with positive signals were colocalized with GFAP positive cells (Fig. 3F; a–e). A small amount of cells with human mitochondria marker were sustained for 16 weeks in the QA+transplant group (Fig. 3G; a–d), indicating that hBM-MSCs could survive *in vivo* for a long period of time. There was no cell with human mitochondria marker detected in the QA−lesioned group (Fig. 3G; e). These findings suggest that some transplanted hBM-MSCs could survive and differentiate into astrocytes and neurons. In addition, some astrocytes encircled the core of the QA−impaired region and the hBM-MSCs attracted microglia nearby. This suggests that hBM-MSCs may differentiate differently and increase the neurogenesis within the QA−impaired striatum.

### hBM-MSCs Improve Survival Rates and Cell Differentiation after Transplantation into R6/2-J2 HD Mice

**Figure 4:**
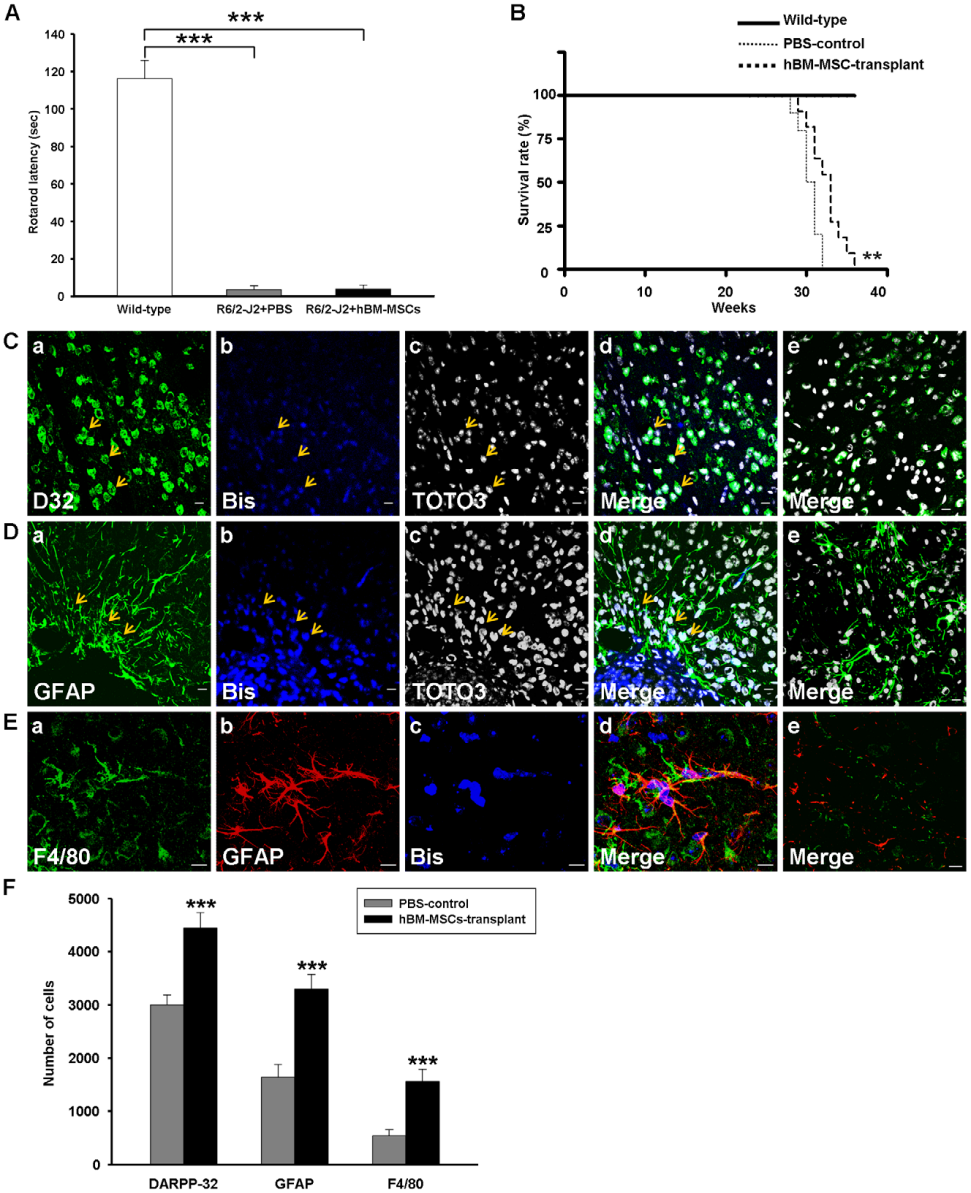
Transplanted hBM-MSCs improved animal survival rate and cell differentiation in R6/2-J2 mice. (A) Rotarod performance of mice after treatment. Compared to the normal motor function exhibited by the wild-type sham-control mice, both the hBM-MSC-transplant and PBS-control R6/2-J2 mice showed significant reduction in latency during the rotarod task. No improvement in motor function was identified in the hBM-MSC-transplant group compared with the PBS-control R6/2-J2 mice at 28 weeks of age. (B) Survival rates of animals after hBM-MSC transplantation. All the wild-type sham-control mice (n = 12) were alive at 36 weeks; whereas, the PBS-control R6/2-J2 mice (n = 10) and the hBM-MSC-transplant R6/2-J2 mice (n = 11) were all died by 32 and 36 weeks of age, respectively. The median survival of PBS-control and hBM-MSC-transplant R6/2-J2 mice were 30.5 and 33 weeks old, respectively, and there was a significant difference between the groups. (C–E) Confocal laser microscope observation of immunofluorescence labeled striatal sections in R6/2-J2 mice, 12 weeks after hBM-MSC transplantation. Transplanted hBM-MSCs pre-labeled with bisbenzimide (C-b, D-b & E-c; blue) or co-labeled with TOTO3 (C-c & D-c; white) were immunostained against cell markers DARPP-32 (C-a; green), GFAP (D-a; green & E-b; red), and F4/80 (E-a; green). Corresponding merged images are shown accordingly (d). Images from the PBS-control R6/2-J2 mice (e) were stained with the same cell markers as the hBM-MSC-transplant group (d). Arrows show the colocalization of hBM-MSCs and cell markers. Scale bar = 10 µm. (F) Quantitation of DARPP-32, GFAP, and F4/80-positive cells in the hemispheres of striata from hBM-MSC-transplant and PBS-control R6/2-J2 groups. Error bars represent SD, and ** *P*<0.001, *** *P*<0.0001.

In order to prove the universal therapeutic potential of hBM-MSCs, a genetically-modified HD rodent model R6/2-J2 was used to investigate the neuroprotective function of hBM-MSCs. Sixteen weeks after surgery, both hBM-MSC-transplant and PBS-control groups showed obvious reduction in rotarod performance compared to the normal mice, but there were no significant differences between hBM-MSC-transplant and PBS-control groups (Fig. 4A). In addition, there were no remarkable difference in striatum volumes between the two HD groups (data not shown); this was perhaps because the extent of striatal atrophy in R6/2-J2 mice was not obvious. These results were not consistent with the effect observed in QA mice and revealed that the severe atrophy in R6/2-J2 mouse muscles could not be rescued efficiently by the transplanted hBM-MSCs in the brain. Although the PBS-control R6/2-J2 mice and the hBM-MSC-transplant R6/2-J2 mice could not survive beyond 32 and 36 weeks of age, respectively, the median survival age of PBS-control (30.5 weeks) and hBM-MSC-transplant R6/2-J2 mice (33 weeks) were significantly different (Fig. 4B). Further investigation of the effects of transplanted hBM-MSCs in the R6/2-J2 mice suggested a similar result to that shown in C57/B6 mice. A portion of the bisbenzimide-labeled cells identified in the striatum of the R6/2-J2 mice retained DARPP-32 expression (Fig. 4C; a–d). Moreover, some of the transplanted hBM-MSCs also emitted intense GFAP signals (Fig. 4D; a–d) throughout the transplanted striatum of R6/2-J2 mice. Some F4/80-expressing cells close to the GFAP-expressing cells were also observed (Fig. 4E; a–d). Such cell differentiation was seldom detected in the PBS-control R6/2-J2 mice (Fig. 4; C-e, D-e & E-e). The quantitative results show a significantly higher number of DARPP-32, GFAP, and F4/80-positive cells in the hBM-MSC-transplant group than in PBS-control R6/2-J2 mice (Fig. 4F). These findings suggest that hBM-MSCs could improve animal survival and cell differentiation not only in the QA-induced mouse model but also in the genetic model of HD.

### Transplanted hBM-MSCs Play a Trophic Role in the HD Mouse Model

**Figure 5:**
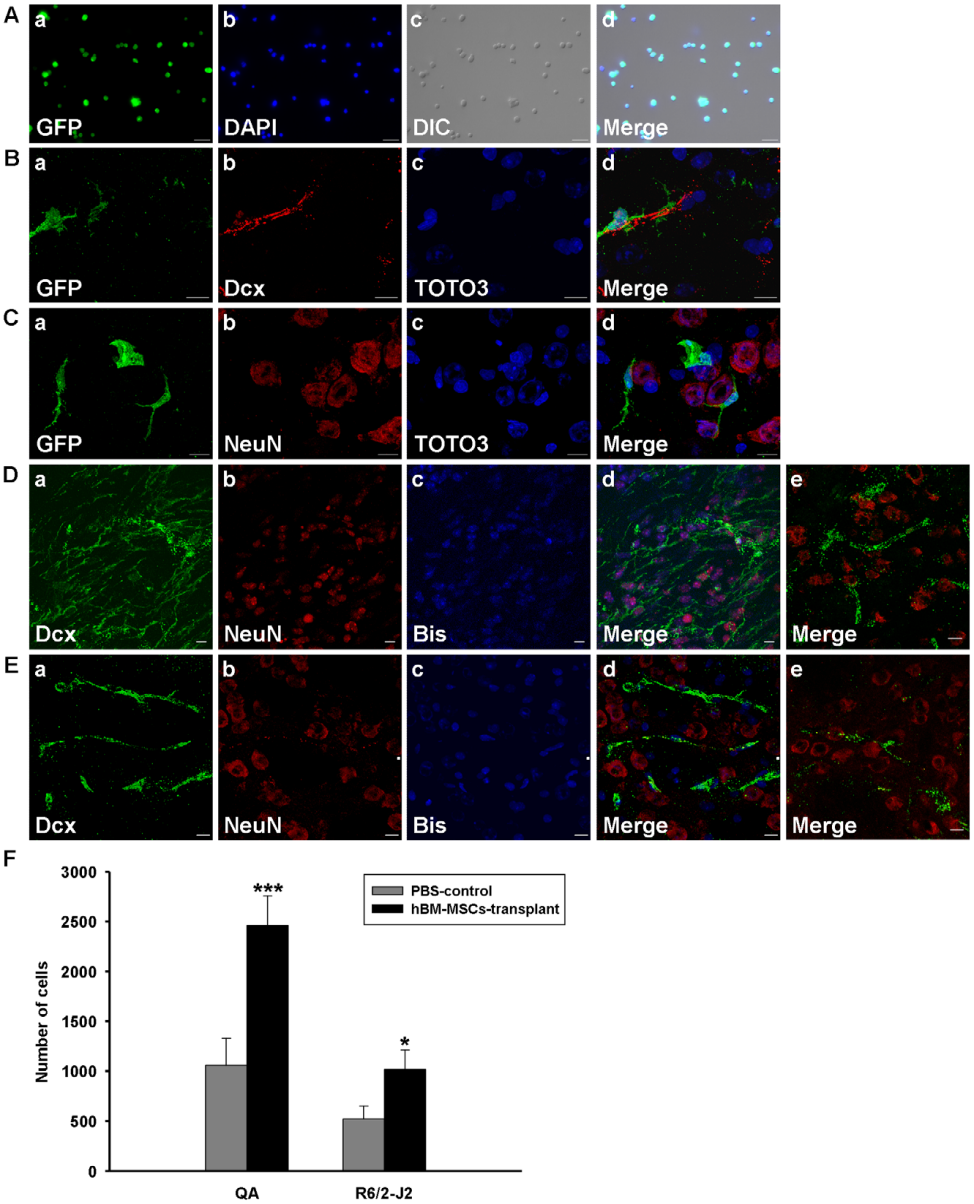
Transplanted hBM-MSCs attract cell migration into the striatum. (A) Confocal laser microscopic observation of cells from mouse bone marrow 8 weeks after bone marrow replacement. Cells were observed under GFP fluorescence (a; green), DAPI nuclear staining (b; blue) and DIC (c) conditions, respectively. Merged images are shown accordingly (d). Scale bar = 20 µm. (B–C) Confocal laser microscopic observation of immunofluorescence labeled striatal sections 8 weeks after hBM-MSC transplantation in mice with QA−lesioned and bone marrow replacement. Bone marrow-derived cells labeled with GFP (a; green) were close to neuroblast cells (B-b; red) or internal neuron cells (C-b; red) in the striatum after hBM-MSC transplantation. TOTO3 signals (c; blue) represent the cell nuclei in the striatum. Corresponding merged images are shown accordingly (d). Scale bar = 10 µm. (D–E) Confocal laser microscopic observation of immunofluorescence labeled striatal sections in hBM-MSCs transplanted QA-lesioned mice (D), 8 weeks after striatal transplantation with hBM-MSCs, or R6/2-J2 mice (E), 12 weeks after striatal transplantation with hBM-MSCs. Transplanted hBM-MSCs pre-labeled with bisbenzimide (c; blue) were immunostained against cell markers, Dcx (a; green) and NeuN (b; red). Corresponding merged images are shown accordingly (d). Images from the sham-control group (e) were stained with the same cell markers as the hBM-MSC-transplant group (d). Scale bar = 10 µm. (F) Quantitation of Dcx-positive cells in the hemispheres of striata in the QA−lesioned and R6/2-J2 mice. Error bars represent SD, and * *P*<0.05, *** *P*<0.0001.

Although only a minority of hBM-MSCs differentiated and expressed different cell markers, including GFAP, NeuN, and DARPP-32, the improvement in survival rate in hBM-MSC transplant mice was significant. We were therefore interested in whether the therapeutic potential of hBM-MSCs was caused by trophic support. Thus, bone marrow replacement mice were used to investigate whether the hBM-MSCs attracted cells from other sources into the striatal regions in QA−lesioned mice. The replacement bone marrow cells were GFP-labeled to distinguish whether the cells attracted by hBM-MSCs came from peripheral blood. After bone marrow replacement, mice underwent QA lesioning and hBM-MSC transplantation as previously described. Immunostaining with GFP antibody showed that all the GFP-derived BM cells had GFP signals eight weeks after BM replacement (Fig. 5A, a–d), and some GFP-expressing neuron–like cells appeared in the striatum (Fig. 5B&C, a–d). Additionally, neuron-like cells were seen in the striatal region, but these neuron-like cells were only found near neuroblasts (marked by Dcx, Fig. 5B; a–d) or neurons (Fig. 5C; a–d) and did not differentiate into these cell-types. In addition, only about 40 GFP-positive cells were identified in the hemispheres of striata of bone marrow-replaced QA−lesioned mice. These results suggest that only a few endogenous bone marrow-derived cells may be attracted by hBM-MSCs to the impaired striatum in QA+transplant mice.

### hBM-MSC Transplantation Induces Migration of Neuroblast Cells in HD Mouse Models

Most GFAP and F4/80 expression was not colocalized with bisbenzimide signals, as described above, so we presumed that the bisbenzimide-unlabeled cells came from the source other than the QA−damaged cells. Having established that after neuronal injury, neural stem cells could replace the dead cells in the brain, we tried to find out whether the bisbenzimide-unlabeled cells were originally endogenous neural stem cells. We found intense staining by Dcx antibody (a marker for mouse neuroblasts) in the QA+transplant group (Fig. 5D; a–d) and R6/2-J2+transplant group (Fig. 5E; a–d). The Dcx-immunoreactive cells were all bisbenzimide-negative cells located close to bisbenzimide-labeled cells which coexpressed NeuN or GFAP. This phenomenon was much less apparent in the PBS-control groups (Fig. 5; D-e & E-e), as shown in the quantitative results (Fig. 5F). Fig. 5 B and C demonstrate that in both the QA-lesioned and genetically-damaged striatum, hBM-MSCs had a trophic effect, and the BM is one of the regions that is affected by the trophic effect. In a previous study, implantation of human MSCs stimulated proliferation, migration, and differentiation of the endogenous neural stem cells within the hippocampus [16]. We hypothesize that GFP negative cells from other sources such as neural stem cells, were attracted by hBM-MSCs in BM replacement mice as shown in Fig. 5 D and E.

### Transplanted hBM-MSCs Might Integrate with Host Cells and Improve Angiogenic Activity in the Damaged Striatal Region

**Figure 6:**
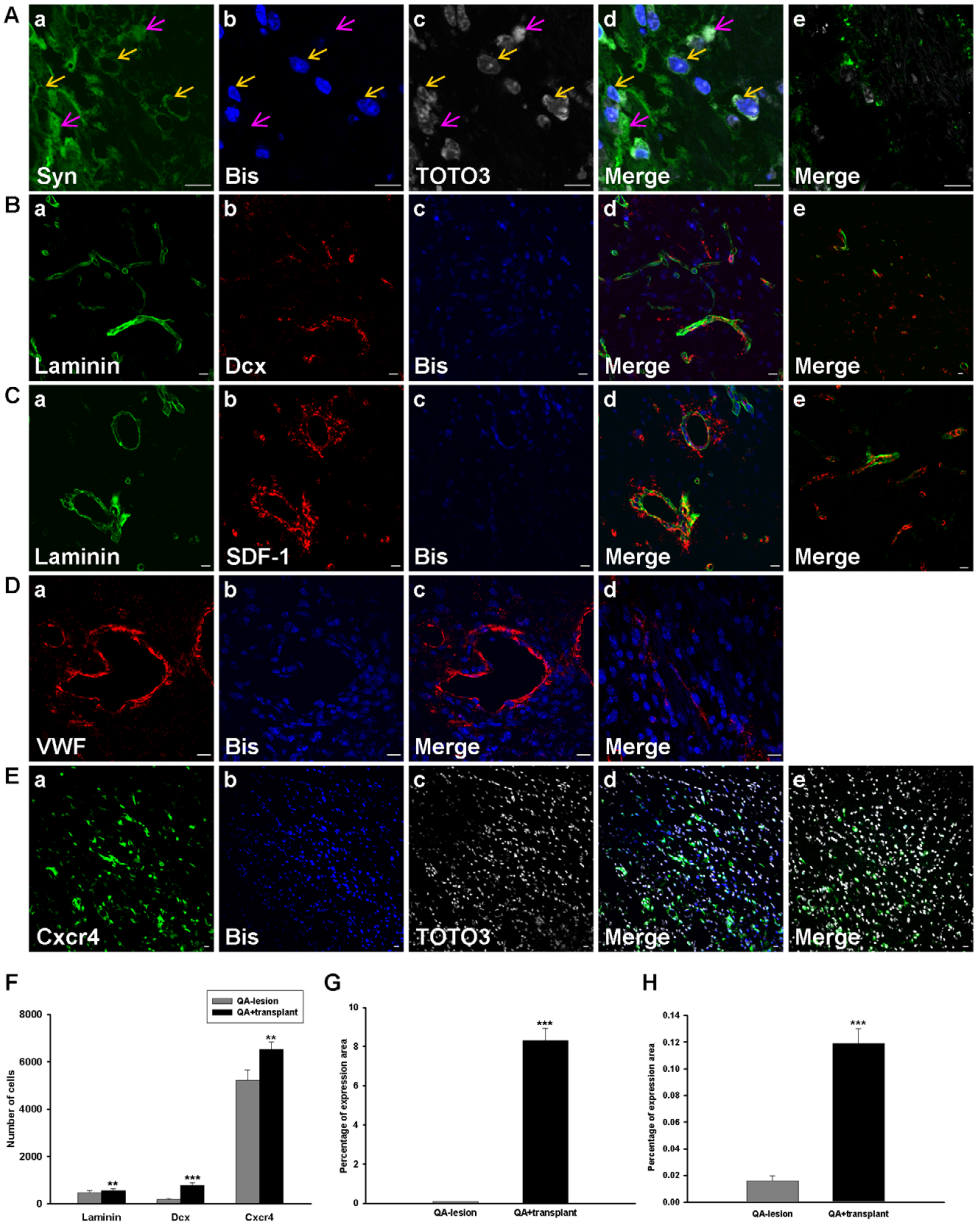
hBM-MSCs raised neural stem cell migration and SDF-1 secretion in QA-lesioned mice. (A–E) Confocal laser microscopic observation of immunofluorescence labeled striatal sections after 8 weeks transplantation of hBM-MSCs into QA−lesioned mice. Transplanted hBM-MSCs pre-labeled with bisbenzimide (A-b, B-c, C-c, D-b & E-b; blue) or co-labeled with TOTO3 (A-c & E-c; white) were immunostained for cell markers, synaptophysin (A-a; green), laminin (B-a, C-a; green), Dcx (B-b; red), SDF-1 (C-b; red), VWF (D-a; red), and Cxcr4 (E-a; green). Corresponding merged images are shown accordingly (A-d, B-d, C-d, D-c & E-d). Images A-e, B-e, C-e, D-d, and E-e were from the QA−lesioned group stained with the same cell markers as A-d, B-d, C-d, D-c & E-d, respectively, except that bisbenzimide labeling was substituted with DAPI staining in D-d. Arrows indicate colocalization of cells and cell markers, yellow for both bisbenzimide and TOTO3 and pink for TOTO3 only. Scale bar = 10 µm. Quantitation of laminin, Dcx, and Cxcr4-positive cells (F) and percentage of SDF-1 (G) and VWF (H) expression areas in the hemispheres of striata from QA+transplant or QA−lesioned groups. Error bars represent SD, and ** *P*<0.001, *** *P*<0.0001.

It has been reported that the raised expression of synaptic marker proteins, including the presynaptic vesicle protein, synaptophysin, is evidence of synapse formation [17], [18]. Our examination showed that synaptophysin expression was colocalized with bisbenzimide-labeled cells (Fig. 6A, a–d). In addition, we also observed that some synaptophysin signals did not colocalize with the bisbenzimide-labeled signals but rather surrounded bisbenzimide-labeled cells (Fig. 6A, a–d) which was seldom detected in the QA-lesioned group (Fig. 6A; e), revealing that hBM-MSCs have the potential to form synapses and might integrate with the host cells.

Since angiogenesis is a prerequisite for tissue reconstitution, hBM-MSCs may improve angiogenesis as they attract neural stem cells and bone marrow-derived cells to migrate to the striatum. Laminin, an extracellular matrix (ECM) marker used to identify the formation of new blood vessels [19] was used here as a marker for angiogenic activity. After hBM-MSC transplantation, intense laminin expression was seen near the hBM-MSC injection region, close to Dcx-expressing areas (Fig. 6B; a–d) but expression was much less in the QA−lesioned group (Fig. 6B; e).

Although the neurogenic effects of hMSCs by increasing neurotrophin signaling have been demonstrated [14], we expected that hBM-MSCs may also exert benefical effects through other pathways. SDF-1 is an indispensable chemoattractant for neuron migration in different brain regions [20]. Laminin is an important ECM that enhances the chemotactic activity of SDF-1 in the thymus [21]. We thus investigated the relationship between laminin and SDF-1 in our QA-lesioned HD mouse model. We did indeed find numerous SDF-1 signals adjacent to the regions of increased laminin expression (Fig. 6C; a–d) around the hBM-MSC injection site, but again, significantly less in the QA−lesioned group (Fig. 6C; e).

We also used the endothelium marker VWF to verify the angiogenic activity after hBM-MSC transplantation. There were intense VWF expression signals in the QA injection region, close to the bisbenzimide-labeled cells, in the transplant group (Fig. 6D; a–c), but not in the QA−lesioned group (Fig. 6D; d). Thus, hBM-MSCs appear to play a trophic role, inducing Dcx and SDF-1 expression in the striatum of transplant mice to improve angiogenesis.

Because the SDF-1 expression was raised after hBM-MSC transplantation, we investigated the expression of the SDF-1 receptor Cxcr4 around the surgical regions. Immunofluorescence staining results show more intense Cxcr4 expression signals around the bisbenzimide-labeled cells in the QA+transplant group (Fig. 6E; a–d) than in the QA−lesioned group (Fig. 6E; e). The quantitative results of the immunofluorescence staining show the numbers of laminin-, Dcx- and Cxcr4-positive cells (Fig. 6F), and the expression areas of SDF-1 (Fig. 6G) and VWF (Fig. 6H) are significantly higher in the QA+transplant group than in the QA−lesioned group. Thus, hBM-MSCs may minimize QA damage through stimulating SDF-1 and Cxcr4 expression.

**Figure 7:**
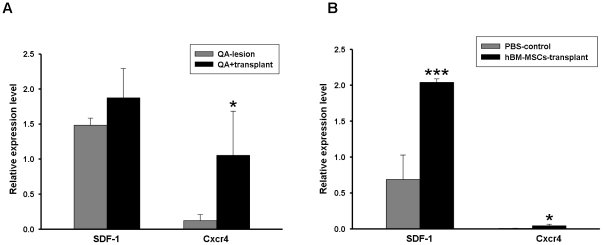
Gene expression levels after transplantation were assessed by qRT-PCR. (A–B) Relative gene expression levels of SDF-1 and Cxcr4 in the brain tissues from QA (A) and R6/2-J2 (B) mice. The relative expression levels of genes were normalized with housekeeping genes; Cxcr4 was normalized with Gapdh, and SDF-1 was normalized with GAPDH. Error bars represent SD, and * *P*<0.05, *** *P*<0.0001.

To further quantify RNA expression after hBM-MSC transplantation, we used qRT-PCR to identify the levels of SDF-1 and Cxcr4. In the QA mouse model 8 weeks after hBM-MSC transplantation, the expression levels of Cxcr4 were increased but the levels of SDF-1 were not significantly different from the non-transplant group (Fig. 7A). Moreover, in the R6/2-J2 mouse model, one week after hBM-MSC transplantation, the expression levels of SDF-1 and Cxcr4 were upregulated (Fig. 7B). These results were similar to the data from immunohistochemical analysis.

### Transplanted hBM-MSCs Reduce Apoptosis after Transplantation in QA−lesioned Mice

**Figure 8:**
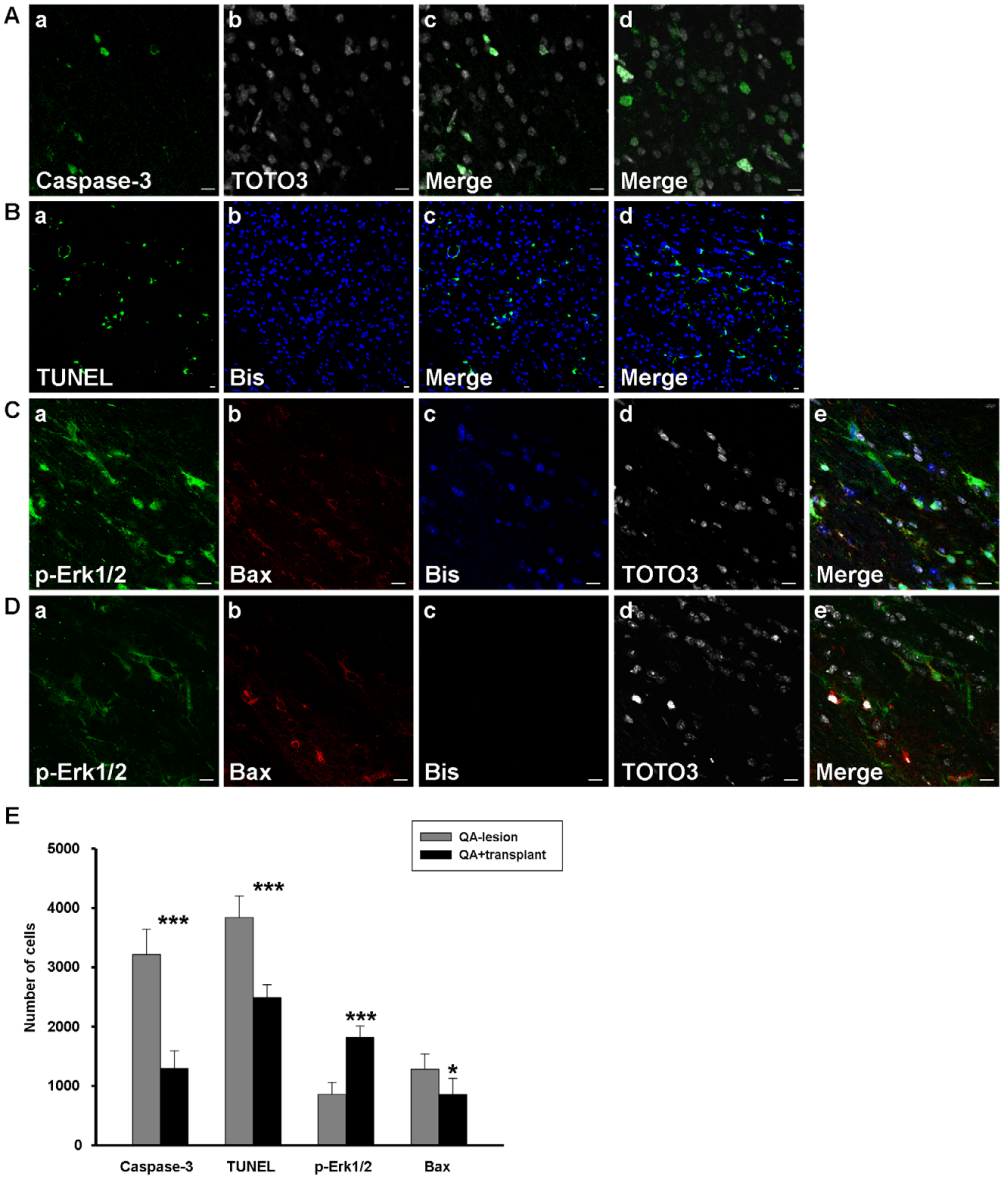
hBM-MSCs reduced apoptosis in QA-lesioned mice. (A–D) Confocal laser microscopic observation of immunofluorescence labeled striatal sections 8 weeks after hBM-MSC transplantation in QA-lesioned mice. Brain slides from the group with transplanted hBM-MSCs pre-labeled with bisbenzimide (B-b & C-c; blue) or co-labeled with TOTO3 (A-b & C-d; white) and from the QA−lesioned group (A-d, B-d & D) were immunostained for the cell markers caspase-3 (A-a; green), TUNEL (B-a; green), p-Erk1/2 (C-a & D-a; green), and Bax (C-b & D-b; red). Corresponding merged images are shown accordingly (A-c, B-c & C-e). Images A-d, B-d, and D are from the QA−lesioned group, stained with the same cell markers as A-c, B-c, and C, respectively, except that the bisbenzimide labeling was substituted with DAPI staining in B-d. Scale bar = 10 µm. (E) Quantitation of caspase-3, TUNEL, p-Erk1/2, and Bax-positive cells in the hemispheres of striata from QA+transplant or QA−lesioned groups. Error bars represent SD, and * *P*<0.05, *** *P*<0.0001.

SDF-1 is constitutively expressed in many tissues and has been demonstrated to significantly inhibit serum depletion-induced apoptosis [22] and downregulate caspase-3 cleavage [23]. We assayed caspase-3 and TUNEL to measure apoptosis after hBM-MSC transplantation, and found that hBM-MSCs decreased both caspase-3 expression (Fig. 8A; a–c) and the level of fragmented DNA (Fig. 8B; a-c) more than in QA−lesioned group without transplantation (Fig. 8; A-d & B-d).

There are many factors involved in apoptotic regulation. In particular, p-Erk1/2 and Bax are two factors that can be modulated by SDF-1 [22], [24]. We found that p-Erk1/2 expression was increased (Fig. 8C; a) and the number of Bax-positive cells was decreased (Fig. 8C; b) in the QA+transplant group (Fig. 8C; a–e) compared to the QA−lesioned group (Fig. 8D; a–e). Both p-Erk1/2 upregulation and Bax downregulation are beneficial in reducing apoptosis. The quantitative results show a significantly lower number of caspase-3, TUNEL, and Bax-positive cells but higher number of p-Erk1/2-positive cells in the QA+transplant group than in QA−lesioned group (Fig. 8E). We thus suggest that hBM-MSCs may diminish the amount of apoptosis through regulation of the expression of p-Erk1/2 and Bax.

## Discussion

**Figure 9:**
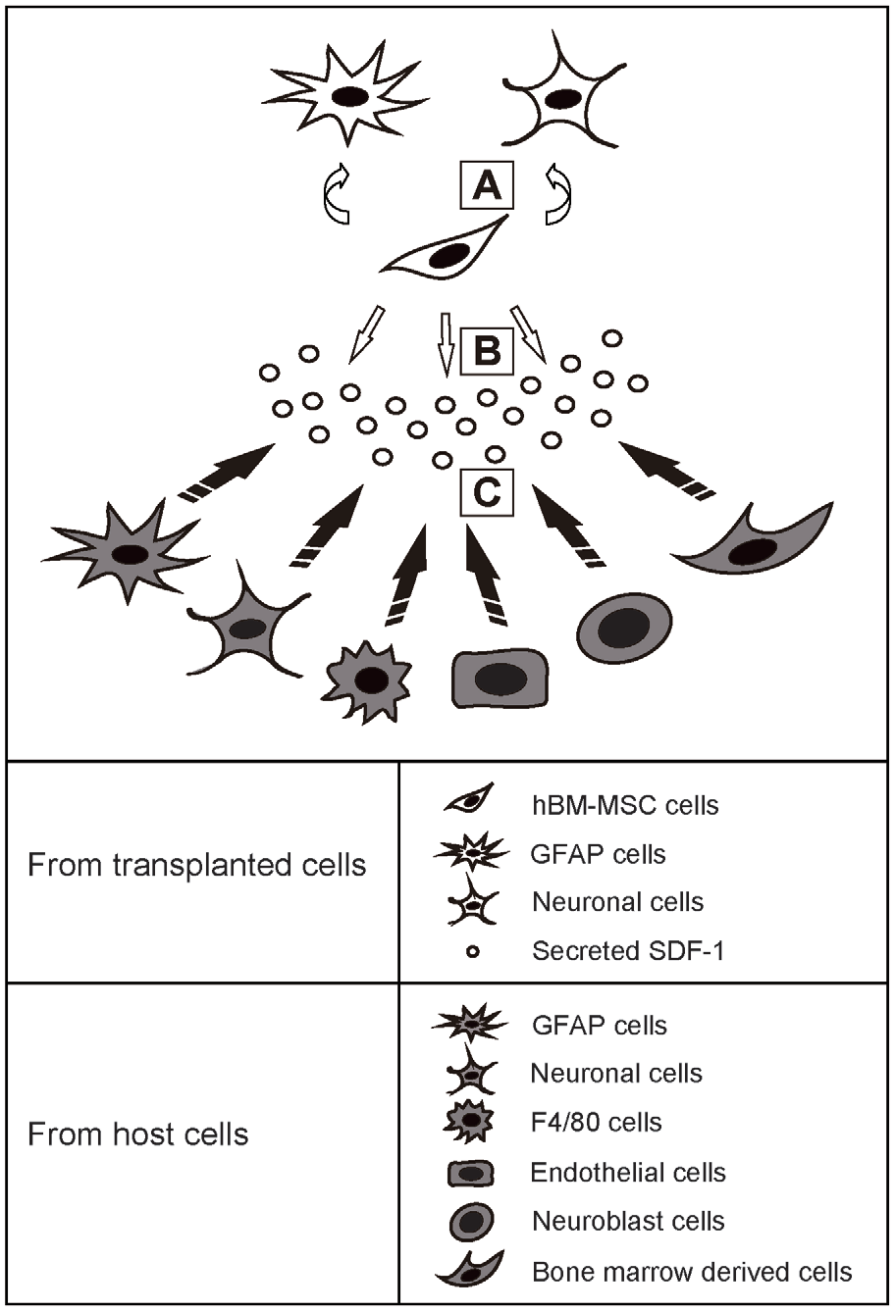
Summary of possible mechanisms by which hBM-MSCs may alleviate cell damage. (A) Transplanted hBM-MSCs may transdifferentiate into GFAP or neuronal cells. (B) The hBM-MSCs may secrete SDF-1 to induce the migration of host cells. (C) Cells near the transplanted hBM-MSCs may undergo proliferation and differentiation to benefit the damaged striatal region.

In the present study, we investigated whether hBM-MSCs can survive, differentiate, and integrate into the striatum after transplantation, and thereby reduce ongoing striatal damage and improve functional recovery in QA−lesioned or genetically-modified HD mouse models. Transplantation of hBM-MSCs reduced motor dysfunction in striatal QA−lesioned mice, as determined by rotarod performance. Within the damaged striatum, a few hBM-MSCs underwent differentiation, as shown by the expression markers for striatal medium spiny projection neurons, mature neurons and astrocytes; and moreover, the stem cells induced microglia and neuroblasts to migrate to the damaged striatal region. These observations were all reproduced in the R6/2-J2 mouse model, except for the amelioration in motor dysfunction. Furthermore, endogenous bone marrow-derived cells were induced by the transplanted hBM-MSCs to approach the QA−lesioned region. The levels of the angiogenesis markers laminin and VWF and of the chemokine SDF-1 near the hBM-MSCs were all elevated. Together, these results indicate that transplanted hBM-MSCs may alleviate cell damage in the HD mouse model through three possible mechanisms: (A) neuronal differentiation, (B) chemokine secretion, and (C) proliferation induction (See the schematic representation in Fig. 9).

Mesenchymal stem cells (MSCs) are a distinguishable population of cells that are able to differentiate into bone, cartilage, and other tissues [25], and can be easily obtained and autotransplanted. Therefore, MSCs have great potential for clinical application. In addition, these cells are not thought to induce strong immunoresponses in recipients [26], and their potential use in therapy for neurodegenerative diseases, such as HD, has been reported [27], [28]. The chemotactic signals and homing capabilities of MSCs are well-proven [29], but the particular mechanisms and molecules involved are still unclear. Some recent studies focusing on the transplantation of MSCs in different HD models have highlighted the potential of MSCs in the amelioration of striatal degeneration [30] and improving behavioral abnormalities in QA−lesioned rats [31]. Furthermore, MSC-transplantation combined with neurotrophic factor secretion showed therapeutic effects in different HD models [28], [32]. Moreover, the function of MSCs in providing trophic support has been demonstrated in a Parkinson's disease model [33]. Therefore, we tried to investigate the details of hBM-MSC application in HD therapy using QA and transgenic mouse models.

The excitotoxicity generated by QA has been demonstrated to induce the degeneration of striatal neurons, with a specific loss of GABAergic neurons [34], and to cause similar abnormalities in motor, emotional, and cognitive functions that appear in HD patients [35]. The QA−lesioned rat model had been used extensively in studies of cell therapy [27], [28], [30], [36], but QA−lesioned mouse models are rare [14], [37]. The application of MSCs to HD transgenic mice has, however, been proved beneficial in increasing neurotrophic factor expression in HD mice [14]. We thus included the QA mouse model as one of our therapeutic targets in our investigations of the possible mechanisms involved in MSC transplantation. On the other hand, the use of a transgenic mouse model such as R6/2-J2 demonstrated the feasibility of application of this research in HD patients with a genome bearing a CAG repeat-expanded huntingtin allele. Early death and lack of neuronal degeneration make the use of R6/2 mice difficult in cell transplantation studies, and we therefore used R6/2-J2 mice, which die around 32–36 weeks of age, as the therapeutic target to provide a longer period for evaluation of functional improvement after cell transplantation.

Astrocyte populations in different brain regions have been shown to have important roles in neurogenic support [38]. In HD brains, mutant huntingtin reduces glial glutamate uptake and this dysfunction may be associated with neuronal excitotoxicity [39]. Moreover, mutant huntingtin may affect the production of chemokines such as tumor necrosis factor alpha (TNF-α) [40] and neurotrophic factors such as glial-derived neurotrophic factor (GDNF) [41] and nerve growth factor (NGF) [42] from glial cells. In our experiments, the small number of hBM-MSCs that differentiated into glial cells may contribute to chemokine secretion and hBM-MSC transplantation may provide a beneficial environment that attracts glial cell activation and proliferation [43]. Once the numbers of activated glial cells are boosted, their trophic support will slow the excitotoxic damage to GABAergic neurons in the striatum.

In the injured brain, some upregulated environmental elements may account for the migration of endogenous neural stem cells or transplanted immortalized, neonate-derived neural precursor cells to the lesioned region and their differentiation into neurons [44], [45]. In the present study, brain injury induced neurogenesis and enhanced neuronal migration to the lesioned region to enhance proliferation of correct cell types, such as Dcx-expressing neuroblasts, to reconstruct the damaged cell architecture, as seen after stroke [46]. It has been demonstrated that Dcx-positive cells can migrate toward the lesioned striatum from the dorsal region of the striatum following neuronal environment disruption, such as from cortical lesions [47], and these cells are in close spatial relationships with blood vessels. Dcx was considered to be a marker for new-born neurons [48] which undoubtedly reflects the number of proliferating cells which were BrdU-positive. In other HD mouse models such as R6/1 and R6/2, diseased mice had decreased neurogenesis [48], [49]. In our studies, Dcx-positive cells were induced by hBM-MSC transplantation, resulting in increased neurogenesis and the recruitment of numerous appropriate cells to further benefit the damaged cells in the striatum.

Laminin, produced by neurons and astroglia, is important in regulation of cell behavior in the central nervous system [50] and vascular remodeling in angiogenesis [19]. Laminin is also important in axon growth and guidance. The identified stem cell niches, such as the subventricular zones (SVZ) [51] and bone marrow stroma, contain laminins [52], thus a close relationship between laminin and stem cells is expected. Bone marrow cell transplantation has been demonstrated to induce angiogenesis [53] and benefit artery disease [54], [55]. Combining these results with our present study, we hypothesize that hBM-MSCs act as a paracrine stimulator and improve laminin expression, resulting in angiogenesis. Such angiogenesis should result in chemokines such as SDF-1 [56], angiogenic molecules, such as vascular endothelial growth factor (VEGF) [57], and neurotrophic factors, such as fibroblast growth factor (FGF), ciliary neurotrophic factor (CNTF), and nerve growth factor (NGF) [14] having more chance to reach the injured region through the newly formed blood vessels, thus further benefiting the damaged neurons, and finally causing the functional improvement seen in our HD mouse models. SDF-1 activates the Cxcr4 receptor expressed in several neuronal cells, including microglia, astrocytes [58], and GABAergic precursors [20], to direct the migration of new neurons [59] and promote neuronal progenitor motility [60] toward damaged neurons. Once laminin improves blood vessel formation, SDF-1 may be secreted and moved through the angiogenic areas to influence neurogenesis.

The mechanisms by which hBM-MSC transplantation benefits QA−lesioned and genetically-manipulated mouse models of HD were investigated in this study. BM-MSC downregulation of the caspase-3-mediated apoptotic pathway in rat spinal cord injury [61] and SDF-1 regulation of anti-apoptosis in different models [22], [23] have been reported. Our study showed that expression of SDF-1 was upregulated by hBM-MSCs, which exerted anti-apoptotic effects through modulation of p-Erk1/2, caspase-3 and Bax, ultimately protecting the QA−lesioned and genetically-blemished cells. In addition, increased neurogenesis and neuroprotection by neurotrophic or growth factors, and new synapse formation with reorganization, have been suggested by stroke models [62]. The decreased striatal atrophy and the improved functional recovery of the HD model in our study also suggest that hBM-MSC transplantation protects the host brain from further destruction. Our results indicate that, within the impaired striatum, while these transplanted hBM-MSCs were viable for at least 120 days after transplantation, only a few transplanted cells (less than 10% of the transplanted hBM-MSCs) actually underwent engrafting, differentiating into neurons and glia. This suggests that transplanted hBM-MSCs at least partially contribute to improved neurological function through the release of various molecules rather than through the establishment of a new neuronal network between the transplanted and the host tissue. Transplanted cells in the excitotoxic lesion have been described to exert trophic support by the secretion of different neurotrophic factors, such as NGF and epidermal growth factor (EGF) [63], [64]. Thus, a few of the neurons surviving in the QA−lesioned and genetically impaired striata or the neurotrophic factors released from the active astrocytes may provide a suitable environmental niche for transplanted hBM-MSCs to settle and further differentiate into neurons or in turn release other neurotrophic factors to induce neurogenesis.

Several clinical studies have used different kinds of tissues as sources for transplantation into the striatum of HD patients, but therapeutic results have not always been predictable [6], [65], [66], [67], [68], [69]. Only Krystkowiak and Reuter found clinical improvement, and this occurred over a period of several years [65], [67]. One possible explanation for these results is that severely damaged tissues surrounding the transplanted cells were unable to protect against degeneration [69]and there was a lack of connection between grafts and transplanted areas that blocked the therapeutic benefits of grafts [67]. In our studies, we found abundant laminin and VWF expression which could provide better connections between grafts and neuronal cells in degenerating regions which might have benefits in cell transplantation. We, therefore, believe that hBM-MSCs may have more potential than the fetal grafts used in previous studies.

In summary, we have demonstrated that hBM-MSCs transplanted intrastriatally survive after transplantation in the QA and genetically impaired striatum, induce functional recovery and improve neuronal differentiation with a proportion of newly-generated cells expressing markers characteristic of neurons or associated cells. Furthermore, we have demonstrated that transplantation of hBM-MSCs reduces the motor function impairment observed in the QA−lesioned HD model. The motor function improvement in this study is promising and lends support to the hypothesis that some transplanted hBM-MSCs have the potential to generate functionally mature striatal neurons that form appropriate graft-host relationships which may restore impaired striatal circuits. Angiogenesis, chemo-attraction, and anti-apoptosis may be the major mechanisms provided by hBM-MSCs. Collectively, this study demonstrates the therapeutic potential of hBM-MSCs for cell replacement therapy and indicates that hBM-MSCs may provide an alternative cell source for transplantation therapy in the treatment of HD and other neurodegenerative diseases.

## Materials and Methods

### Animal Subjects

Experimental procedures involving animals were approved by the Animal Care and Use Committee of the Institute of Molecular Biology, Academia Sinica with permit number RMiIMBLH2008041, and all the experimental procedures involving animals were and performed according to the guidelines established by the Animal Care and Use Committee of the Institute of Molecular Biology, Academia Sinica, Taipei, Taiwan. Ninety adult 8- or 12-week-old male C57/B6 mice weighing 25–30 g (National Laboratory Animal Center, Taipei, Taiwan) and sixty 12-week-old male R6/2-J2 mice (The Jackson Laboratory) were used in this study. R6/2-J2 mice are a R6/2 line with an average of 298 CAG repeats in a mixed CBA/J and C57BL/6J background. All animals were randomly housed, five mice per cage, at 21°C and 65% humidity with a 12-h light/dark cycle (lights on at 07:00 am). Water and food were freely available throughout the study.

### Bone Marrow Replacement Mice

Adult 8-week-old male C57/B6 mice were anesthetized with an intraperitoneal (i.p.) injection of 0.5 g/kg chloral hydrate (Riedel-de Haen, Seelze, Germany), and received 1200 rad total body γ-irradiation (Mark I Model 68A Irradiator, J.L. Shepherd and Associates, CA). Twenty-four hours later, γ-exposed mice were transplanted with 3×10^6^ bone marrow cells obtained from femurs of 12-week-old GFP transgenic mice by tail vein injection. Eight weeks later, the bone marrow replacement mice underwent excitotoxic lesioning.

### Cell Culture of hBM-MSCs

Adult hBM-MSCs were kindly provided by Dr. Junya Toguchida (Kyoto University, Japan). The cells were immortalized to a cell-line by retrovirus-mediated gene transfer of human telomerase reverse transcriptase (hTERT) combined with human papillomavirus E6 and E7 without affecting their potential for adipogenic, osteogenic, and chondrogenic differentiation. The cell-line was maintained in DMEM (GibcoBRL) with 10% FBS (GibcoBRL) and 100 U/ml penicillin/streptomycin (GibcoBRL). On the day of transplantation, hBM-MSCs were labeled with bisbenzimide (Bis; 1 µg/ml; Sigma) 10 min before detachment with trypsin.

### Excitotoxic Lesioning and hBM-MSC Transplantation into Mouse Striatum

Before surgery, 12-week-old male C57/B6 mice were anesthetized (0.5 g/kg chloral hydrate i.p.) and positioned in a stereotaxic apparatus (Stoelting). All mice received unilateral intrastriatal injections of 1 µl quinolinic acid (QA) dissolved in 0.1 M phosphate buffer (PBS) (85 nmole/µl, pH 7.4) via a 10 µl Hamilton syringe at the following coordinates: AP +0.5 mm, ML −2.0 mm, and DV −3.0 mm, from the bregma. Seven days post QA administration mice were randomly divided into two groups. One group of mice received a unilateral transplantation of hBM-MSCs at the QA-lesioned striatum, while the second group of mice received a unilateral injection of PBS at the corresponding site. In 12-week-old R6/2-J2 mice, hBM-MSCs or PBS vehicle was transplanted into the right hemisphere of the striatum. hBM-MSCs were transplanted into two adjacent sites in the striatum (∼100,000 viable cells/µl; 2 µl per injection site) at the following stereotaxic coordinates: site 1 = AP 0 mm, ML −2.0 mm, and DV −3.0 mm DV, from the bregma; site 2 = AP +1.0 mm, ML −2.0 mm, and DV −3.0 mm, from the bregma. The syringe was left in place for 2 min before injection of cells over 5 min and was retracted after an additional 5 min.

### Rotarod Performance

An accelerating rotarod for mice (Model 47600, Ugo Basile) was used to test motor performance in QA−lesioned mice, QA+transplant mice, hBM-MSC-transplant R6/2-J2 mice, PBS-treated R6/2-J2 mice, and age and sex-matched sham-control littermates. Twice a week during the experimental period, three trials of 120 sec each at 30 r.p.m. for the QA mouse model and 16 r.p.m. for the R6/2-J2 mouse model were performed per day.

### Bromodeoxyuridine Labeling

Bromodeoxyuridine (BrdU), a thymidine analogue which is incorporated into the DNA of dividing cells during S-phase, was used for mitotic labeling of proliferating cells (Sigma). The three groups of mice in this study (QA−lesioned, QA+transplant, and sham-control) received daily injections of BrdU (50 mg/kg i.p.) for 7 consecutive days before sacrificing.

### Histologic analysis

Histologic analysis of mouse-brain volumes was performed as previously described [70]. Continuous 50 µm coronal brain sections from the striatum regions (bregma 1.54 to 0.10 mm) in the neostriatum were used for volumetric analysis. The areas of the striatum as determined by Nissl staining were calculated from each continuous section and total volumes were measured by integrating each section area and depth using Image-Pro Plus 6.0 software (Media Cybernetics, MD, USA).

### Immunocytochemistry

Immunocytochemistry was performed to examine the characteristics of the hBM-MSCs in the brains of mice that received surgery. Mice were killed by an overdose of chloral hydrate, perfused transcardially with chilled PBS and fixed with 4% paraformaldehyde (pH 7.4 in PBS). Brains were removed, postfixed overnight at 4°C, cryoprotected by immersion in 15% sucrose solution for one day and transferred to 30% sucrose solution for another day. Coronal sections (8 µm) were cryocut through the striatum of frozen brains, mounted on poly-L-lysine coated slides (Sigma), and stored at −20°C for immunocytochemistry.

For BrdU immunostaining, DNA was first denatured by incubating each brain section in 50% formamide in 2× standard saline citrate at 65°C for 2 h, immersed in 2 N HCl at 37°C for 30 min, and finally rinsed in 0.1 M boric acid (pH 8.5). Sections were then rinsed with Tris buffer and incubated with 1% H_2_O_2_ to block endogenous peroxidase. Immunostaining was performed using the labeled streptavidin-biotin method (DAKO LASB-2 Kit, Peroxidase, DAKO). Brain slides were incubated with the appropriate diluted antibodies to BrdU (for nuclear identification, 1∶400; Boehringer Mannheim) at room temperature for 1 h. The slides were then washed with Tris-buffered saline containing 0.1% Tween-20, and the specimens were sequentially incubated for 10–30 min with biotinylated anti-rabbit (1∶200; R&D Systems) immunoglobulins and peroxidase-labeled streptavidin. Staining was performed after 10 min incubation with a freshly prepared substrate-chromogen solution and then counterstained with hematoxylin. Brightfield images were taken using a digital camera on a light microscope (Olympus IX70) using the SPOT software system (Diagnostic Instruments, Sterling Heights, MI).

Immunofluorescence was performed on mounted sections. After rinsing briefly in PBS, slides were treated with 5% FBS in PBST (PBS plus 0.2% Tween-20) for 30 min, all at room temperature, and were then incubated with the primary antibody in PBST overnight at 4°C. The antibodies used in this study include rabbit anti-DARPP-32 (D32; 1∶200; Chemicon), mouse anti-NeuN (1∶50; Chemicon), rabbit anti-GFAP (1∶300; Chemicon), rat anti-F4/80 (1∶50; Serotec), mouse anti-mitochondria (1∶200; Millipore), goat anti-doublecortin (Dcx; 1∶50; Santa Cruz), mouse anti-synaptophysin (1∶200; Chemicon), rabbit anti-laminin (1∶100; Sigma), goat anti-SDF-1 (1∶100; Santa Cruz), rabbit anti-VWF (1∶100; DAKO), rabbit anti-Cxcr4 (1∶50; Torrey Pines Biolabs), rabbit anti-caspase-3 (1∶100; Cell Signaling), rabbit anti-p-Erk1/2 (1∶100; Chemicon), and mouse anti-Bax (1∶100; BD). Sections were then rinsed three times in PBST and incubated for a further hour with second antibodies, including Alexa Fluor 488, 546, or 647-labeled donkey anti-mouse, anti-rabbit, anti-rat, or anti-goat IgG (1∶500; Invitrogen) antibodies, and Alexa Fluor 642-labeled TOTO-3 iodide dye (1∶1000; Invitrogen) or 4′-6-Diamidino-2-phenylindole (DAPI; 1∶1,000; Sigma) simultaneously to label the positions of cell nuclei. Fluorescent-stained sections were analyzed on a confocal laser-scanning microscope (Carl Zeiss LSM510) equipped with UV, argon, argon/krypton and helium/neon lasers.

### TUNEL Assay

A TUNEL assay was performed to evaluate cell apoptosis after hBM-MSC transplantation. After rehydration, slides were treated with 20 µg/ml proteinase K for 8–10 min at room temperature, washed with PBS, and re-fixed in 4% paraformaldehyde. Once equilibrated, the slides were labeled for the fragmented DNA of apoptotic cells using Nucleotide Mix and rTdT enzyme (DeadEnd fluorometric TUNEL system, Promega,) at 37°C for 60 min and the reaction stopped by incubation in 2× SSC for 15 min. After washing with deionized water, the slides were covered with glass coverslips and analyzed under the fluorescence microscope.

### Cell Counting

Cell counting was undertaken as described previously [62], [71]. Cells were counted in three coronal sections covering the entire striatum in three adjacent areas (AP 0 mm, AP +0.5 mm, and AP +1.0 mm from bregma) close to the hBM-MSC transplantation areas (AP 0 mm and AP +1.0 mm from bregma) in three independent experiments under a microscope at 400× magnification. Unbiased stereological quantification of the total hemispheric areas of each section was made with an image analysis system (Image-Pro Plus 6.0, Media Cybernatics) to determine the number and expression areas of the cells with positive signals.

### RNA Extraction

Brain tissues were obtained from mice at different time-points, and total RNA was extracted using Trizol reagent (Invitrogen). About 100 mg of brain tissue was homogenized with 1 ml of Trizol and 200 µl of chloroform and then shaken to mix. The reaction was kept at room temperature for 5 min and spun for 15 min at 13,000 g at 4°C. The upper phase was then collected and mixed with 500 µl of isopropanol to precipitate the RNA and spun for 10 min at 12,000 rpm at 4°C. The RNA pellet was then dissolved in 50 µl of diethyl pyrocarbonate (DEPC) -treated water and quantified by spectrophotometer using a Narodrop (Biolab) and kept for quantitative Real Time RT-PCR (qRT-PCR).

### Quantitative real time RT-PCR Amplification

Quantitative real time RT-PCR (qRT-PCR) was performed using SsoFast EvaGreen Supermix kit (Bio-Rad). Commercially designed primers (Bio-Rad) for SDF-1 (NCBI: NM_000609) and Cxcr4 (NCBI: NM_009911) were applied for gene expression quantification; therefore, all primer sets possessed high genetic specificity and produced a single dissociation and melting (Tm) curve. Table 1 shows the sequences of primer sets used and the annealing temperatures were all set at 55°C. One microgram of total RNA isolated from brain tissues was used in a 20 µl cDNA synthesis reaction using an iScript cDNA Synthesis kit (Bio-Rad). One microliter of tenfold diluted cDNA sample was applied to the PCR analysis within a total reaction volume of 20 µl. qRT-PCR amplification was conducted with MiniOption System (Bio-Rad); the thermal profile consisted of 30 sec at 98°C followed by 40 cycles of 2 sec at 98°C and 5 sec at 55°C, and then an additional 5 sec annealing step at 65°C. The relative quantification (ddCt) data was normalized with the corresponding housekeeping genes, Gapdh (NCBI: NM_008084) or GAPDH (NCBI: NM_002046), in order to calculate the relative levels of gene expression by the comparative method (Bio-Rad).

**Table 1:** Primer sequences used in qRT-PCR experiments.

Accession number	Sequence definition	Forward primer	Reverse primer
NM_009911	Cxcr4	GGACTGTAGAACTGTAGAG	AACCAAACAAACCATCAC
NM_000609	SDF-1	ATTAGAGATTACCTCCTGAGAA	GGTCCAATGAGATCCAATG
NM_008084	Gapdh	ACCTGCCAAGTATGATGA	GGAGTTGCTGTTGAAGTC
NM_002046	GAPDH	GGTCGGAGTCAACGGATT	GGCAACAATATCCACTTTACCA

### Statistical Analysis

All values shown in the figures are presented as mean ± SD. One-way ANOVA in combination with Bonferroni's post-hoc analysis were used to evaluate the Rotarod performance and survival rates for unequal samples sizes. The numbers and the expression areas of immunopositive cells, and the differences in gene expression levels between groups measured by qRT-PCR were analyzed by Student's *t*-test for unpaired samples, using Prism 4.0 (GraphPad software, CA, USA). A value of *P*<0.05 was considered statistically significant.
